# Community acquired infections in older patients admitted to hospital from care homes *versus* the community: cohort study of microbiology and outcomes

**DOI:** 10.1186/1471-2318-13-12

**Published:** 2013-02-06

**Authors:** Charis Marwick, Virginia Hernandez Santiago, Colin McCowan, Janice Broomhall, Peter Davey

**Affiliations:** 1 Division of Population Health Sciences, Medical Research Institute, University of Dundee, Mackenzie Building, Kirsty Semple Way, Dundee, DD2 4BF, Scotland, UK; 2 Robertson Centre for Biostatistics, Institute of Health and Wellbeing, University of Glasgow, Boyd Orr Building, Level 11, Glasgow, G12 8QQ, Scotland, UK

**Keywords:** Long-term care facilities, Nursing home, Care home, Sepsis, Severity of infection, Antibiotic policy

## Abstract

**Background:**

Residents of care homes are at risk of colonisation and infection with antibiotic resistant bacteria, but there is little evidence that antibiotic resistance among such patients is associated with worse outcomes than among older people living in their own homes. Our aim was to compare the prevalence of antibiotic resistant bacteria and clinical outcomes in older patients admitted to hospital with acute infections from care homes *versus* their own homes.

**Methods:**

We enrolled patients admitted to Ninewells Hospital in 2005 who were older than 64 years with onset of acute community acquired respiratory tract, urinary tract or skin and soft tissue infections, and with at least one sample sent for culture. The primary outcome was 30 day mortality, adjusted for age, sex, Charlson Index of co-morbidity, sepsis severity, presence of resistant isolates and resistance to initial therapy.

**Results:**

161 patients were identified, 60 from care homes and 101 from the community. Care home patients were older, had more co-morbidities, and higher rates of resistant bacteria, including MRSA and Gram negative organisms resistant to co-amoxiclav, cefuroxime and/or ciprofloxacin, overall (70% *versus* 36%, p = 0.026). 30 day mortality was high in both groups (30% in care home patients and 24% in comparators). In multivariate logistic regression we found that place of residence did not predict 30 day mortality (adjusted odds ratio (OR) for own home *versus* care home 1.01, 95% CI 0.40-2.52, p = 0.984). Only having severe sepsis predicted 30 day mortality (OR 10.09, 95% CI 3.37-30.19, p < 0.001), after adjustment for age, sex, co-morbidity, presence of resistant bacteria, resistance to initial therapy, and place of residence.

**Conclusions:**

Older patients admitted with acute infection had high 30 day mortality. Patients from care homes were more likely to have resistant organisms but high levels of antimicrobial resistance were found in both groups. Thus, we recommend that antibiotic therapies active against resistant organisms, guided by local resistance patterns, should be considered for all older patients admitted with severe sepsis regardless of their place of residence.

## Background

Residents of care homes are at risk of colonisation and infection with antibiotic resistant bacteria [1], especially meticillin resistant *Staphylococcus aureus* (MRSA) [2] and cephalosporin or quinolone resistant Gram negative bacteria [3,4]. A study from Ohio showed that the incidence of MRSA infection was increasing faster in nursing home residents than in hospital inpatients. In comparison with 2006 the incidence of MRSA in 2007 increased among nursing home residents by 183% whereas the increase for inpatients was only 58% [5]. There are several factors that may explain the increasing prevalence of antibiotic resistant bacteria in care homes. These include the fact that they are a closed environment which might facilitate the spread of infection. Residents are usually older, multi-morbid and vulnerable, and likely to have indwelling devices. They are frequently admitted to hospitals, with a greater risk of colonisation with, and transmission of, resistant organisms from acute care facilities [1]. In addition, 50-75% residents in long-term care facilities are exposed to at least one course of antibiotics each year [6].

The objective of this study was to compare the prevalence of antibiotic resistance and clinical outcomes in older patients admitted with acute infections from care homes *versus* the community using a retrospective cohort design.

## Methods

### Cohort definition

We conducted a retrospective cohort study. The study population consisted of residents of the Tayside region in Scotland aged 65 or over in 2005 who were admitted to Ninewells Hospital between 1^st^ January and 31^st^ December 2005, had at least one sample sent for culture, and were either; i) discharged with an ICD 10 code for lower respiratory tract infection (LRTI), skin and soft tissue infection (SSTI), or urinary tract infection (UTI), or ii) identified opportunistically during screening for related cohort studies. Ninewells Hospital is an 850-bed teaching hospital serving a population of approximately 400,000 people.

We identified study subjects by searching the Scottish Morbidity Records database of hospital admissions (SMR01) for admissions of patients over 64 years old in 2005 with pre-specified ICD 10 discharge codes. The ICD 10 discharge codes used were J13-15, J18.1, J18.9, J22 for lower respiratory tract infections; L031, L97X, M6008, M86, R02X, S913, T131, T814, T874 for skin and soft tissue infections; N309, N390 for urinary infections. Case notes were obtained and patients were included if the presence of infection was documented, and the patient received antibiotic treatment, within three days after admission to hospital.

Additional study subjects who met the above criteria, with the exception of the ICD 10 discharge codes, were identified opportunistically during case note screening for two related cohort studies; one study of patients with intra-abdominal infections, and one of patients who had blood cultures taken but had no ICD 10 discharge codes for infection.

### Data sources

The Health Informatics Centre (HIC) is a partnership between NHS Tayside, NHS Fife and the University of Dundee. It allows linkage of individual patient data from different electronic datasets, including hospital admission data (Scottish Morbidity Record, SMR01), mortality data (through the National Register of Deaths, GRO), demographic data, microbiology data (from Central Vision, the information system that contains the results of all laboratory and radiology investigations performed in NHS Tayside) and prescribing information from community pharmacies.

### Data extraction, linkage and analysis

The exposure variable was residence in a nursing or residential home, collectively termed care homes. The addresses for all nursing and residential homes were taken from Health Board records and any patient who was registered at one of these addresses was identified as a care home resident.

The primary outcome was death, in or outside hospital, within 30 days of admission with infection. This was ascertained from the National Register of Deaths. Covariates for the mortality analysis were place of residence, sepsis severity, resistance of isolates, age, sex and Charlson Index of co-morbidity [7].

A Charlson Index of co-morbidity was calculated for each individual from hospital discharge codes for the current admission and any other admissions in the previous year. Case note reviews were performed to extract details about evidence of site (LRTI, SSTI, UTI or other) and severity of infection. We used a previously published method to stratify severity of infection according to markers of sepsis (presence of the systemic inflammatory response syndrome in the context of infection) [8] and severe sepsis (Standardised Early Warning System score ≥4) [9].

Microbiological information including sensitivity data were obtained from Central Vision.

Data about initial antimicrobial therapy and results of all microbiology tests were reviewed independently by two Infectious Diseases physicians (CM and PD) in order to address three questions about each microbial isolate from cultures:

1. Was the isolate clinically significant?

2. If significant, was it a “resistant” pathogen, i.e. meticillin resistant *Staphylococcus aureus* (MRSA), or Gram negative bacteria resistant to cefuroxime, co-amoxiclav and/or ciprofloxacin?

3. If significant, was it sensitive to at least one antimicrobial included in the initial therapy?

Decisions about the clinical significance of each microbial isolate were made depending on the site of culture. Blood cultures: all isolates were coded as clinically significant with the exception of coagulase-negative staphylococci. Skin: only *Staphylococcus aureus* (*S. aureus*) isolates were coded as clinically significant, as there were no streptococci isolated from any skin samples in the study. Sputum: *Streptococcus pneumoniae, S. aureus, Haemophilus influenzae, Moraxella catarhalis, Klebsiella spp. Pseudomonas spp.* and *Stenotrophomonas maltophilia* were classified as clinically significant, and other isolates (e.g. *Escherichia coli* (*E. coli*) or other coliforms) were classified as not significant. Urine: all isolates were coded as clinically significant.

### Statistical analysis

Proportions or means with 95% confidence intervals (CI) were reported as appropriate. Differences in proportions between groups were examined using the chi-squared test, and means using the student *t*-test, with the test result, degrees of freedom and p-value reported. Odds ratios (OR) for 30 day mortality were calculated by univariate and multivariate logistic regression models adjusted for age, gender, co-morbidity, severity of sepsis, presence of a resistant organism, resistance to initial therapy and place of residence. All data were analysed in Stata Version 10 (StataCorp. 2007. *Stata Statistical Software: Release 10*. College Station, TX: StataCorp LP).

### Ethics

Ethics approval was not required for this study as we used data from electronic and paper hospital records and followed the HIC Standard Operating Procedure, which is pre-approved by the NHS Research Ethics Committee. The protocol was approved by the Caldicott Guardian for hospital inpatients.

## Results

We identified 161 valid cases of community acquired infections in patients aged 65 years and over in 2005 with at least one sample sent for microbiology. Of these, 101 (63%) were resident in their own homes and 60 (37%) were in care homes. Residents of care homes were significantly older (*χ*^2^ = 14.95, df = 3, p = 0.002) and had significantly higher Charlson co-morbidity indices (*χ*^2^ = 6.07, df = 2, p = 0.048) than patients who were resident in their own homes (Table 1). The most common co-morbidities were cerebrovascular disease, diabetes, chronic pulmonary disease and myocardial infarction.

**Table 1:** Demographic details, infection site and microbiological isolates

**Demographic variables**	**Own home**	**Care home**	**p value (*X***^**2**^**test)**	**Total**
No. of patients	101	60		161
Age (years): 65-69	18 (18%)	6 (10%)	0.002	24 (15%)
70-79	44 (44%)	12 (20%)		56 (35%)
80-89	31 (31%)	34 (57%)		65 (40%)
90+	8 (8%)	8 (13%)		16 (10%)
Male gender	49 (49%)	25 (42%)	0.399	74 (46%)
Charlson Index*: 0	24 (24%)	9 (15%)	0.048	33 (20%)
1-2	61 (60%)	32 (53%)		93 (58%)
3^+^	16 (16%)	19 (32%)		35 (22%)
Site of infection **				
LRTI	29 (29%)	13 (22%)	0.199	42 (26%)
SSTI	20 (20%)	12 (20%)		32 (20%)
UTI	24 (24%)	20 (33%)		44 (27%)
Other	23 (23%)	8 (13%)		31 (19%)
Multiple	5 (5%)	7 (12%)		12 (7%)
Microbiological isolates				
No. of patients	22	23		45
*S. aureus*	11 (50%)	12 (52%)	0.884	23 (51%)
MRSA	4 (18%)	10 (43%)	0.067	14 (31%)
Gram negative	10 (45%)	12 (52%)	0.652	22 (49%)
Resistant Gram negative ***	4 (18%)	8 (35%)	0.208	12 (27%)
Any resistant organism	8 (36%)	16 (70%)	0.026	24 (53%)
Resistant to initial therapy	8 (36%)	13 (57%)	0.175	21 (47%)

*Charlson Index of comorbidity [7].

**LRTI, lower respiratory infection; SSTI, skin and soft tissue infection; UTI, urinary tract infection; Other infections include intra-abdominal infections, bone/joint infections, and bacteraemia with undefined source.

***Resistant Gram negative bacteria were resistant to cefuroxime, co-amoxiclav or ciprofloxacin. Two patients had both MRSA and resistant Gram negative bacteria.

The most common sites of infection were the lower respiratory tract for patients admitted from their own home and the urinary tract for patients admitted from care homes (Table 1), but the overall distribution of sites of infection was similar for both groups of patients (*χ*^2^ = 6.00, df = 4, p = 0.199). Forty five patients had 62 clinically significant bacterial isolates; 23 patients (38%) from care homes and 22 patients (22%) from their own home (*χ*^2^ = 5.12, df = 1, p = 0.024) (Table 1). The commonest organisms isolated were: *S. aureus* and *E. coli*. Patients from care homes were significantly more likely to have MRSA if *S. aureus* was isolated (10/12 (83%) *versus* 4/11 (36%) isolates, respectively, (*χ*^2^ = 5.32, df = 1, p = 0.02)). A higher proportion of care home residents had resistant Gram negative organisms but the difference was not significant. Having any resistant organism was significantly more common among care home residents (Table 1).

Twenty four of 45 (53%) patients received antimicrobial therapy that did not cover all the organisms isolated from that patient. This was more common among care home residents (61%) than those from their own home (45%), but the difference was not statistically significant (*χ*^2^ = 1.07, df = 1, p = 0.300). Of the 24 patients with isolates that were resistant to their initial antimicrobial therapy, the isolates were MRSA in 14 (58%) cases, and Gram negative bacteria resistant to cefuroxime, co-amoxiclav and/or ciprofloxacin in 7 (29%) cases. In the remaining three cases the isolates resistant to initial therapy were; amoxicillin sensitive *Enterococcus faecalis* treated with cefuroxime, meticillin sensitive *S. aureus* treated with amoxicillin, and penicillin sensitive *S. pneumoniae* treated with ciprofloxacin. The 24 isolates that were resistant to empirical therapy came from blood (11 isolates), skin (7 isolates), urine (4 isolates) and sputum (2 isolates). All of these isolates were sensitive to the combination of amoxicillin, gentamicin and metronidazole.

Indicators of sepsis or severe sepsis were present in 41 (41%) and 15 (15%) patients resident in their own home, and 21 (35%) and 18 (30%) patients resident in care homes, respectively, with no significant difference between the groups (*χ*^2^ = 5.36, df = 2, p = 0.069). Significant organisms were isolated from nine (27%) of the 33 patients with severe sepsis. The organism was MRSA in four (12% of 33) cases, *Morganella morganii* in two (6%) cases, and *Streptococcus pneumoniae*, *Klebsiella pneumoniae*, *Pseudomonas aeruginosa*, and *Escherichia coli*, each in one case. Three of the Gram negative isolates were classified as resistant organisms, and one patient had both MRSA and a resistant *Morganella morganii*, meaning that overall six (18% of 33) patients with severe sepsis had resistant organisms. This was not significantly different from the 14% (18/128) of patients without severe sepsis who had resistant organisms (*χ*^2^ = 0.35, df = 1, p = 0.554).

There were 42 (26%) patients who died within 30 days of admission to hospital, 24 (24%) among those resident in their own home, and 18 (30%) resident in care homes, with no significant difference between the groups (*χ*^2^ = 0.76, df = 1, p = 0.383). Among those who died, 10 (24%) had resistant organisms isolated, 3/24 (13%) admitted from their own home and 7/18 (39%) admitted from care homes (*χ*^2^ =3.95, df = 1, p = 0.070). There was no difference between mean lengths of stay (t = −0.058, df = 157, p = 0.953) among patients admitted from care homes (14.8 days, 95% CI 9.4-19.8) and from their own home (14.6 days, 95% CI 10.5-19.2). In univariate analysis the only significant predictor of 30 day mortality was having severe sepsis (OR 8.60, 95% CI 3.21-23.02, p < 0.001, compared to no sepsis), and this was even stronger (adjusted OR 10.09, 95% CI 3.37-30.19, p < 0.001) after adjustment for age, sex, co-morbidity, resistance of organisms and place of residence in multivariate regression (Table 2). We found that place of residence did not predict 30 day mortality (adjusted OR for own home *versus* care home 1.01, 95% CI 0.40-2.52, p = 0.984). Isolation of resistant bacteria was associated with higher mortality but the differences were not statistically significant (Table 2).

**Table 2:** Odds ratios with 95% CI and p-values for 30 day mortality by variable*

**Variable**	**Odds ratios for death within 30 days**	
	**(95% CI and p value)**	
	**Unadjusted**	**Adjusted**
Own home (*versus* care home)	1.38 (0.67 - 2.82, p = 0.384)	1.01 (0.40 - 2.52, p = 0.984)
Sepsis (*versus* no sepsis)	1.85 (0.74 - 4.64, p = 0.192)	1.75 (0.67 - 4.59, p = 0.254)
Severe sepsis (*versus* no sepsis)	8.60 (3.21 - 23.02, p < 0.001)	10.09 (3.37 - 30.19, p < 0.001)
Any resistant organism (*versus* no resistant organism isolated)	1.89 (0.76 - 4.72, p = 0.172)	2.10 (0.26 - 16.77, p = 0.486)
Resistant to initial therapy (*versus* not resistant)	1.92 (0.73 - 5.02, p = 0.184)	0.64 (0.07 - 5.85, p = 0.696

^*^Patients aged 90+ were at greater risk of 30 day mortality than the youngest age group but otherwise there was no significant effect of age, gender or co-morbidity.

## Discussion

There is growing evidence about the increasing prevalence of multi drug resistant bacteria in care homes or long term care facilities. However, this is the first study to compare the prevalence of resistance and the clinical outcomes of older patients presenting to hospital with infection from care homes *versus* their own homes. We found that care home residents were older and likely to have more co-morbidity. Residence in a long-term care facility was also associated with a higher prevalence of resistant organisms, which might be associated with a greater exposure to antibiotics in this setting. However, severity of sepsis was the only statistically significant determinant of outcome.

A recent study from Australia compared bacterial isolates and antibiotic resistance in elderly patients from care homes and the community but did not measure clinical outcome [10]. Their study population was also restricted to patients aged ≥ 65 years but, unlike our study, they included patients presenting to ambulatory care settings (Accident and Emergency or Outpatients) that were not admitted to hospital. Their results were broadly similar to our study in that there were higher proportions of antibiotic resistance among some bacteria in nursing home patients, especially meticillin resistance among *S. aureus* isolates across all specimen types, and resistance to several empiric antibiotics among Gram negative isolates in urine cultures [10]. A study from the USA compared clinical outcomes in patients with isolation of antibiotic resistant *versus* sensitive bacteria who were transferred from long-term care facilities and admitted to acute-care hospitals, but did not include patients admitted from their own homes [11]. That study included 153 patients with positive microbiology and 42 (27%) received an initial regimen to which their isolate was resistant. As in our study, they found that neither infection with antibiotic resistant bacteria nor appropriateness of initial treatment regimen was significantly related to outcome.

The limitations of our study include those common to all retrospective cohorts, such as case ascertainment bias when using hospital discharge codes. In addition, the clinical data available, including microbiological samples, were limited to that carried out as part of routine care. The number of eligible patients for our study was small which was at least in part due to the relatively small proportion of patients who had microbiological samples taken, and the even smaller proportion with a candidate organism isolated. The small numbers limited the power of the study, especially in adjusted analyses. We were unable to determine the timing of microbiological sampling in relation to the timing of antibiotic administration, so prior antibiotic therapy might have limited the number of positive samples. Studies consistently report that microbiological diagnoses are made in low proportions of septic patients. Even among patients with septic shock in intensive care only 70% had a plausible microbiological isolate [12], and we included patients who did not meet sepsis criteria so the low yield was not surprising. Antibiotic exposure prior to admission, which we did not record, may also have had an effect on resistance on admission to hospital. However, neither the bacteria isolated nor antimicrobial resistance were associated with a significant difference in outcome. We were unable to determine the time to antibiotic therapy, or other aspects of sepsis management such as fluid resuscitation, that may have impacted on patient outcome. An updated, larger, prospective study that incorporates more detailed clinical management data would shed further light on the impact of antibiotic resistance on outcome.

## Conclusions

Older patients hospitalised with acute infection had high 30 day mortality. This was 17% even in patients without sepsis and rose to 64% in patients with severe sepsis. When a candidate organism was isolated, there were high levels of antimicrobial resistance in both care home residents and patients resident in their own home. We recommend that therapies active against resistant organisms, guided by local prevalence and resistance data, should be considered for all older patients admitted with severe sepsis, and that place of residence should not alter the choice of empiric antimicrobial regimen.

## Abbreviations

GRO: General Register Office for Scotland; HIC: Health Informatics Centre, University of Dundee; ICD 10: International Classification of Diseases version 10 (World Health Organisation); LRTI: lower respiratory tract infection; MRSA: meticillin resistant *Staphylococcus aureus*; SMR01: Scottish Morbidity Records of hospital admissions; SSTI: skin and soft tissue infection; UTI: urinary tract infection.

## Competing interests

PD and CM have received funds for research, and CM has received support for conference attendance, from Jansen Cilag. The other authors declare that they have no competing interests.

## Authors’ contributions

CM, PD and CMcC designed and oversaw the study. JB created the study database, and carried out the case note reviews and data collection. CM and PD independently classified all bacterial isolates as described in the text. CMcC did the main data analysis with input from VHS, CM and PD. VHS wrote the first draft of the manuscript. All authors reviewed the manuscript and contributed to the final version.

## Authors’ information

PD and CM are Infectious Diseases physicians and researchers with research interests including antimicrobial stewardship and epidemiology. VHS is an academic general practitioner with an interest in antimicrobial prescribing and resistance. CMcC is a lecturer and JB a research nurse with relevant research skills and experience in health services and population research.

## Pre-publication history

The pre-publication history for this paper can be accessed here:

http://www.biomedcentral.com/1471-2318/13/12/prepub
